# Low Noise Structure Design and Experimental Verification of Ship Based on Flexural Wave Band Gap Characteristics

**DOI:** 10.3390/ma18194615

**Published:** 2025-10-06

**Authors:** Yicheng Lu, Li Tang, Chuanlong Wang, Zilong Peng, Li Xiang

**Affiliations:** 1 College of Energy and Power, Jiangsu University of Science and Technology, Zhenjiang 212100, China; 2 Marine Design and Research Institute of China, Shanghai 200011, China; 3 Hunan Special Equipment Inspection & Research Institute, Changsha 410013, China

**Keywords:** flexural wave, bandgap characteristics, low-noise structures, band gap modulation, acoustic optimization design

## Abstract

To address low-frequency vibration and noise issues in ship grating structures, this study proposes a novel acoustic optimization design method based on modulating flexural wave bandgap characteristics. By establishing an equivalent periodic spring-mass coupled beam model to predict bandgap properties, its effectiveness is validated through numerical simulations and experimental testing. By selectively enhancing longitudinal stiffness while weakening transverse components, the bandgap characteristics are effectively tuned to target frequency bands. This approach achieves an 8.2 dB noise reduction at the 31.4 Hz natural frequency. The results demonstrate that bandgap-based design provides a numerically and experimentally validated solution for low-noise ship structures.

## 1. Introduction

Ships constitute a complex fluid–structure interaction system where internal compartments, armor plates, and hull sides interact with the surrounding fluid, leading to significant energy exchange. Traditional vibration and noise control methods often fail to effectively analyze vibration transmission paths or address the root causes of low-frequency spectral peaks, thus lacking targeted solutions for such noise [1,2]. Recent advances in acoustic metamaterials and phononic crystals have introduced innovative approaches to low-frequency vibration control, including bandgap regulation, acoustic black hole optimization, composite metamaterial design, and intelligent design methodologies.

Substantial progress has been made in bandgap generation and regulation mechanisms. Redondo [3] developed a lattice-corrected equivalent model elucidating the coupling between Bragg and local resonance bandgaps, enabling accurate prediction of hybrid bandgaps in the 50–500 Hz range. Jing [4] designed a hierarchical phononic crystal that increased bandgap width by 40%, covering 50–1000 Hz. Yang [5] proposed a chiral lattice structure utilizing rotational symmetry to achieve vibration attenuation between 100 and 800 Hz. Bao [6] created an acoustic demultiplexer via topological optimization for multi-frequency wave separation. In acoustic black hole research, Bao [7] proposed a triple-gradient structure to achieve noise attenuation in the 50–1500 Hz frequency band, while Xiao [8] validated noise reduction effectiveness of ABH vibration dampers at frequencies above 100 Hz in railway applications. Gao [9] discovered ultra-wide bandgaps in nested ABH (acoustic black holes) structures through evanescent Bloch wave analysis.

Wave-based methods have also seen notable development in ship engineering. Yao [10] applied wave analysis to study flexural wave propagation in ship structures and designed impedance mismatch bases for vibration isolation. Liu [11] investigated wave propagation in periodically stiffened hull plates, establishing a theoretical basis for structural vibration control. Lu [12] used Mindlin plate theory to extract flexural wave dispersion characteristics for defect detection. Sorokin [13] combined boundary integral equations with Floquet theory to model wave propagation in fluid-loaded shells. Ruzzene [14] found that by leveraging the bandgap effect generated by periodic structures, elastic wave propagation is suppressed through periodic impedance mismatch in the core layer material, thereby achieving passive vibration control. Shen [15] integrated functionally graded materials with periodic structures to achieve broadband noise reduction and enhanced vibration attenuation. Kafesaki [16] revealed frequency modulation effects in elastic wave bandgap materials, establishing quantitative relationships between defect states and periodic parameters.

Further innovations include An’s [17] gradient resonator sandwich superstructure, achieving efficient vibration attenuation in the 130–180 Hz frequency band through multi-bandgap coupling mechanisms, Chang’s [18] multilayer metamaterial which achieves high sound insulation performance at low frequencies across a broad bandwidth without significantly increasing weight, Kong’s [19] butterfly-shaped porous metamaterial for low-frequency absorption, Xu’s [20] membrane-based design for underwater applications, Zhang’s [21] ultra-thin tunable absorber using buckling plates, and Chen’s [22] deep learning-optimized metabeams. Wang [23] additionally developed methods for estimating and broadening the bandgap of piezoelectric metamaterials, successfully achieving an increase in bandgap width.

To address low-frequency line spectrum noise in ship grillage structures, this paper proposes a bandgap-based design method using an equivalent periodic spring-mass beam model. The paper is organized as follows: First, an equivalent computational model of a periodic spring-mass coupled beam is established, and a theoretical analysis method for its flexural wave bandgap characteristics is proposed. Subsequently, a low-noise structural design process based on bandgap characteristics is elaborated in detail, covering numerical prediction methods for underwater acoustic radiation, holistic, and localized bandgap design strategies, and its application to ship grating structures. This includes experimental verification of vibration attenuation effects. Finally, the main conclusions drawn from this research are summarized.

## 2. Analysis Method of Wave Characteristics of Ship Grillage Structure

The use of equivalent mechanical models represents a fundamental methodology for analyzing structural mechanical properties. Chen Tieyun [24] pioneered the simplification of hull structures into basic mechanical models including continuous beams and rigid grillages to investigate hydrostatic problems. Building upon this foundation, Kurzhakov [25] made significant advances by developing an analytical solution for low-frequency flexural vibrations in orthogonally reinforced grillages. His approach effectively represented transverse reinforcements as elastic supports with concentrated masses positioned at stiffener intersections, achieving this transformation through modal and vibrational shape similarity principles.

**Figure 1:**
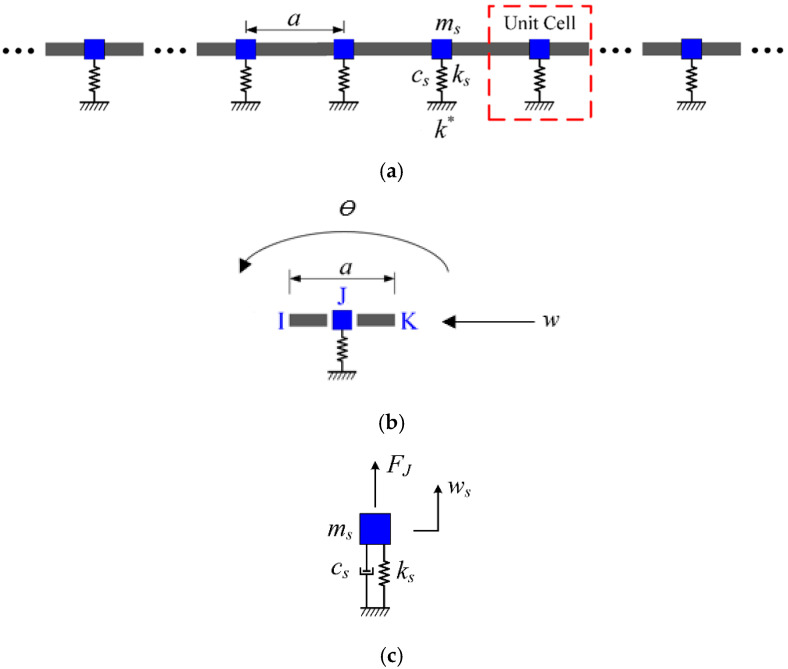
Wave characteristics analysis equivalent computational model of single periodic grillage structure. (**a**) Schematic diagram of a one-dimensional damped single-period spring coupling beam. (**b**) Schematic diagram of the single cell structure of the single-period equivalent calculation model. (**c**) Schematic diagram of beam-mass oscillator coupling system in a single periodic cell.

In this paper, we extend Kurzhakov’s equivalent approach by applying the energy equivalence principle to model the ship’s grillage structure as a periodic spring-mass coupled beam system. The equivalent unit cell Figure 1 consists of two homogeneous beam segments connected in parallel with a spring-mass oscillator at node J, forming a coupled vibration system that neglects the oscillator’s dimensional influence along the beam length.

The structural parameters in Figure 1 include: vibrator spring stiffness $k_{s}$, spring mass $m_{s}$, damping $c_{s}$, lattice constant a, and vertical displacement $w_{s}$; the moment of inertia of the homogeneous beam section I, the section area A, and the supporting stiffness of the elastic foundation $k^{*}$; the material parameters of the beam with periodic spring vibrators include the elastic modulus E, density ρ, Poisson’s ratio v, the translational damping $c_{1}$ and rotational damping $c_{2}$ of the beam, lateral translation degree of freedom w, and rotational degree of freedom θ.

When the equivalent section size (length × width) is much smaller than the length of the beam, the shear deformation of the beam and the moment of inertia of the section about the central axis have negligible effects on the flexural vibration, which is called the Euler–Bernoulli beam. In order to calculate and analyze the vibration characteristics of the beam more accurately, the Timoshenko beam theory takes into account the effects of shear deformation and section moment of inertia. The governing equation for flexural vibration of a homogeneous Timoshenko beam supported by an elastic foundation can be expressed as follows:

(1) $$EI\frac{\partial^{2}\varphi }{\partial x^{2}}+\kappa GA\left(\frac{\partial w}{\partial x}-\varphi \right)-c_{2}\frac{\partial \varphi }{\partial t}-\rho I\frac{\partial^{2}\varphi }{\partial t^{2}}=0$$

(2) $$\kappa GA\left(\frac{\partial^{2}w}{\partial x^{2}}-\frac{\partial \varphi }{\partial x}\right)-c_{1}\frac{\partial w}{\partial t}-\rho A\frac{\partial^{2}w}{\partial t^{2}}+k^{*}w=0$$

where G=E/[2(1+v)] is the shear elastic modulus of the beam material, v is Poisson’s ratio, κ is Timoshenko shear correction coefficient, w and φ represent the vertical and angular displacement of the beam, respectively, and t represents time.

By combining Equations (1) and (2) and eliminating the angular displacement φ, the differential equation of motion for a Timoshenko beam expressed only by the vertical displacement w can be obtained as:

(3) $$\begin{array}{l} \frac{\partial^{4}w}{\partial x^{4}}+\frac{k^{*}}{\kappa GA}\frac{\partial^{2}w}{\partial x^{2}}-\left(\frac{c_{1}}{\kappa GA}+\frac{c_{2}}{EI}\right)\frac{\partial^{3}w}{\partial x^{2}\partial t}-\left(\frac{\rho }{\kappa G}+\frac{\rho }{E}\right)\frac{\partial^{4}w}{\partial x^{2}\partial t}+\left(\frac{c_{1}}{EI}-\frac{c_{2}k^{*}}{EI\kappa GA}\right)\frac{\partial w}{\partial t}\\ +\left(\frac{\rho A}{EI}+\frac{c_{1}c_{2}}{EI\kappa GA}-\frac{\rho k^{*}}{E\kappa GA}\right)\frac{\partial^{2}w}{\partial t^{2}}+\left(\frac{\rho c_{2}}{EI\kappa G}+\frac{\rho c_{1}}{EI\kappa G}\right)\frac{\partial^{3}w}{\partial t^{3}}+\frac{\rho^{2}}{E\kappa G}\frac{\partial^{4}w}{\partial t^{4}}-\frac{k^{*}}{EI}w=0 \end{array}$$

According to Timoshenko beam theory, the relation of shear force V and flexural moment M in a beam to vertical displacement w and transfer displacement φ can be expressed as:

(4) $$M=EI\frac{\partial^{2}w}{\partial x^{2}}$$

(5) $$V=\kappa GA\left(\varphi -\frac{\partial^{2}w}{\partial x^{2}}\right)$$

Shear force V and flexural moment M in a simultaneous homogeneous beam, and vertical displacement w and transfer displacement φ are expressed in matrix form to obtain generalized displacement vector $W_{d}$ and generalized force vector $W_{f}$, expressed as:

(6) $$W_{d}=A_{d}P_{h}(-x)a+D_{d}P_{h}(x)d$$

(7) $$W_{f}=A_{f}P_{h}(-x)a+D_{f}P_{h}(x)d$$

The beam–mass coupling system in a single periodic cell of an infinitely long periodic spring oscillator coupled beam is shown in Figure 1c. The relationship between displacement continuity and force balance of the internal components of the periodic cell at node J can be expressed as:

(8) $$W_{d}^{JI}=T_{d}^{J}W_{d}^{JK}$$

(9) $$W_{f}^{JI}=T_{f}^{J}W_{f}^{JK}+F^{J}$$

In the formula, $T_{d}^{J}$ and $T_{f}^{J}$ represent the generalized displacement transformation matrix and generalized force transformation matrix at the J node in a single periodic cell, respectively, and $F^{J}$ represents the reaction force vector of the spring-mass oscillator acting on the beam.

Substituting the expressions of $W_{d}$ and $W_{f}$ into Equations (8) and (9), the scattering relationship at node *J* can be obtained as:

(10) $$A^{J}a^{J}+D^{J}d^{J}=0$$

In the formula, $a={\left\{{\left(a^{IJ}\right)}^{T}{\left(a^{JI}\right)}^{T}{\left(a^{JK}\right)}^{T}{\left(a^{KJ}\right)}^{T}\right\}}^{T}$ and $d={\left\{{\left(d^{IJ}\right)}^{T}{\left(d^{JI}\right)}^{T}{\left(d^{JK}\right)}^{T}{\left(d^{KJ}\right)}^{T}\right\}}^{T}$ are the arrival wave amplitude vector and the departure wave amplitude vector at the node J of a single periodic cell, respectively. A and D are the corresponding coefficient matrices of the arrival wave amplitude vector a and the departure wave amplitude vector d.

According to the generalized displacement and generalized force at the node, combined with the Bloch theorem of periodic structure, the overall scattering relationship of the periodic cell of the infinitely long periodic spring oscillator coupled beam structure is obtained as follows:

(11) Aa+Dd=0

## 3. Low Noise Structure Design of Ship Based on Band Gap Characteristics

The underwater radiated noise of the ship mainly includes mechanical noise, propeller noise, and hydrodynamic noise. Mechanical noise is an important part of the underwater radiated noise of the ship. The underwater radiated noise generated by the vibration of the hull structure excited by the equipment is the main component of the mechanical noise of the ship. The underwater radiated noise caused by the flexural wave propagation in the structure is the main component. In this paper, the band gap characteristics of periodic structure flexural wave are considered to control the low-frequency line spectrum vibration noise and structural design of ship grillage structure.

### 3.1. Numerical Prediction Method of Ship Underwater Acoustic Radiation

The hull structure of a ship can be regarded to some extent as a spatially periodic lattice structure. Equipment excitation is transmitted through the foundation to the hull lattice, inducing a vibration response that propagates as structural waves. Coupling between the hull structure and the surrounding fluid ultimately results in underwater radiated noise.

**Figure 2:**
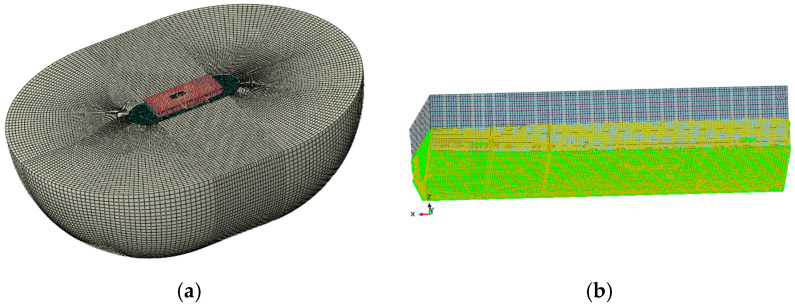
Finite element model for ship cabin structure mechanical vibration analysis. (**a**) Cabin flow field coupling model. (**b**) Finite element model of cabin section.

**Figure 3:**
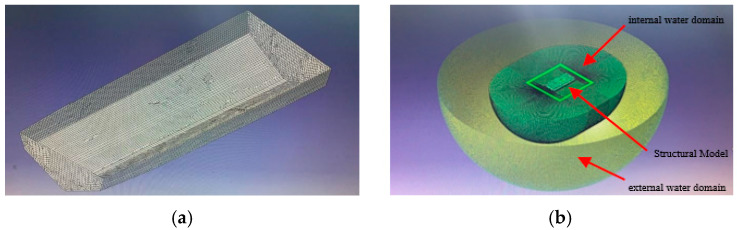
Finite element model of ship cabin structure underwater acoustic radiation analysis. (**a**) Acoustic Grid Model Diagram. (**b**) Underwater Acoustic Radiation Computational Model.

This study uses coupled acoustic–structural numerical methods to simulate low-frequency mechanical noise from ship grillage structures, facilitating the assessment of line spectrum vibrations and the development of noise control strategies. A finite element model of the compartment was built in Abaqus Figure 2, with dimensions of 25 m × 11 m × 6.2 m. The model includes 79,699 grid cells for the structure and internal fluid, while the external water domain is represented as a 25 m-radius hemisphere with 480,157 elements, forming a fully coupled system Figure 3. Boundary conditions include symmetry at the free surface and unit harmonic excitation at equipment–base connections. Harmonic analysis yields the wetted surface vibration response, whose acceleration spectrum serves as input for noise prediction. The mesh size is 0.2 m for the cabin and internal field, and a graded mesh of 0.2–0.7 m, expansion ratio 3.5, is adopted externally to ensure both accuracy and efficiency.

The excitation force load is applied at the connection between the mechanical equipment and the base, and the acceleration spectrum characteristics of the vibration response of the wet surface of the compartment segment are calculated and analyzed.

The indirect boundary element method in Virtual. Lab was employed to calculate sound power and determine the final underwater radiated noise source level. As shown in Figure 3, the analysis model integrates the wet surface where the hull contacts water as an acoustic mesh, while the dry surface where the hull contacts air is defined as a structural mesh and field point mesh. Symmetrical conditions were applied at the waterline to simulate free-surface reflections. Structural vibration results generated by Abaqus (.odb format) were mapped onto the structural mesh, with the wet surface defined as the acoustic mesh. A hemispherical field point mesh with a radius of 100 m and a mesh size of 0.7 m was established, centered at the waterline height Figure 3b. The computational workflow includes transferring wet-surface vibration acceleration from structural analysis to the acoustic mesh to define vibration boundary conditions. This coupling is achieved via Tie constraints or surface-based acoustic coupling.

### 3.2. Overall Design Method of Band Gap

This paper investigates the bandgap and vibration transmission characteristics of periodic ship grillages, where flexural waves reflect, transmit, and transform at structural joints—a process shaped by the skeleton morphology. To compare different structures clearly, we adopt normalized parameters: the horizontal axis represents the frequency normalized to 100 Hz, and the vertical axis gives the base-10 logarithm of acceleration power spectral density relative to a unit reference.

**Figure 4:**
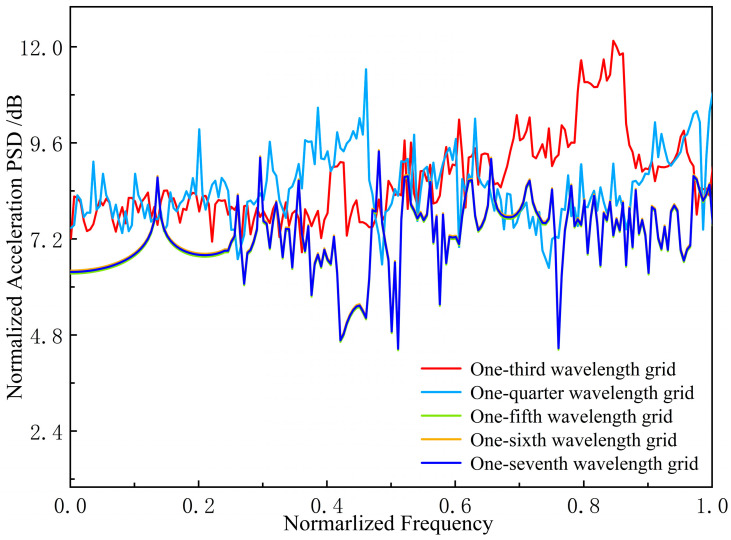
Comparison of grid convergence test results.

To assess simulation accuracy, convergence tests were conducted on a transverse grillage structure. Results were compared across five mesh sizes: one-third, one-quarter, one-fifth, one-sixth, and one-seventh of the wavelength. The comparison results are shown in Figure 4.

The comparison results indicate that beyond the one-fifth wavelength mesh size, further refinement of the mesh has no significant effect on the results. Therefore, the simulation results are judged to have converged.

**Figure 5:**
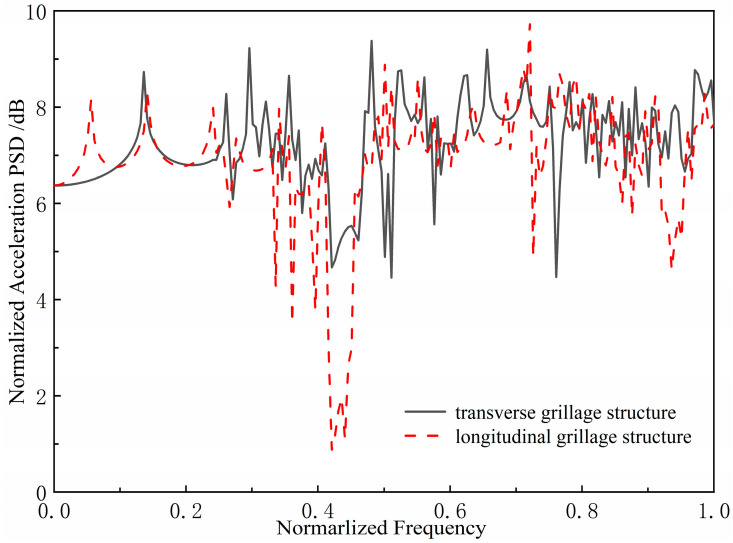
Diagram of power variation with OER (Overall Expansion Ratio) under different currents.

As shown in Figure 5, under unit excitation, the longitudinal grillage produces lower bending vibration levels than the transverse grillage at the assumed scale.

Studies show that flexural waves in ship grillages propagate primarily along the longitudinal direction, with transverse waves remaining as near-field vibrations. Since longitudinal structures behave similarly to periodic spring-mass beams, they exhibit stronger bandgaps and therefore better damping performance. Accordingly, we propose a bandgap-based design approach: on the premise of ensuring structural strength, the longitudinal members are reinforced and the transverse ones weakened, designing the bottom grillage as a longitudinal-skeleton type for overall vibration suppression.

**Figure 6:**
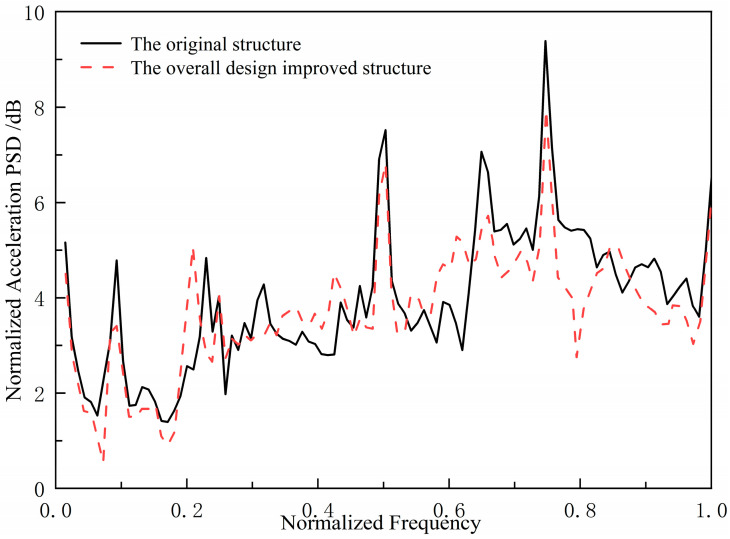
Comparison of underwater acoustic radiation spectrum characteristics between the original structure and the overall design improvement structure.

In order to verify the effectiveness of the design method, a double-bottom grillage structure in the engine room of a certain ship was selected as the research object. An improved structure was constructed by adjusting the arrangement of longitudinal and transverse stiffeners. Excitation was applied to the inner bottom base panel to simulate equipment loads, and the acoustic radiation characteristics of the outer bottom wet surface were measured. The comparison results are shown in Figure 6. The comparison results indicate that the underwater acoustic radiation performance of the improved structure is better than that of the original structure, confirming the effectiveness of the method of optimizing the skeleton layout to control the bandgap characteristics.

### 3.3. Local Optimization Adjustment Band Gap Frequency Band Design Method

The original structure shows two strong line spectrum peaks in the 0.6–0.8 band, while its bandgaps lie mainly below 0.5. We therefore optimized the grillage parameters to shift the bandgaps upward, covering the peaks for effective suppression. Since the bandgap frequency is controlled by the stiffness, k, of the periodic spring oscillator, adjusting k allows the bandgap to be moved to the target higher frequency range, attenuating the line-spectrum peaks.

**Figure 7:**
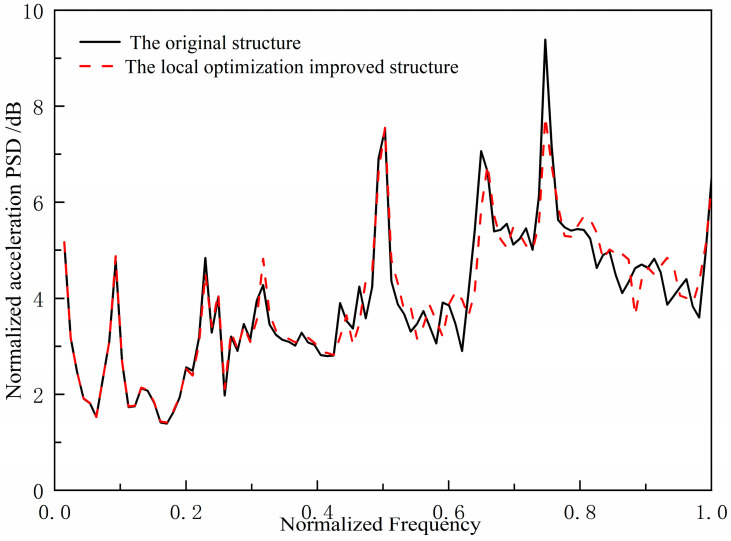
Comparison of underwater acoustic radiation spectrum characteristics between the original design scheme structure and the improved design scheme structure.

On the basis of the original ship double deck grillage structure, the method of enhancing the transverse stiffener stiffness of ship engine room double deck grillage structure is used to optimize the structure locally. By using numerical method, the acoustic source level spectrum curve of the locally optimized and improved structure under unit excitation force is compared with that of the original structure, as shown in Figure 7. Compared with the original grillage structure, the two spectral peaks of the locally optimized and improved structure decreased to a certain extent in the range of 0.6~0.8 band, reaching 2.9 dB and 16.1 dB, respectively. Compared with the original structure, the improved ship grillage structure has better vibration and noise control effect of the low-frequency spectrum. Therefore, the locally optimized and adjusted band-gap frequency band design method is effective.

The above comparative study shows that both the overall band gap design method using the curved band gap characteristics of periodic structure and the local optimization and adjustment band gap frequency band design method are effective. The design ideas of the two low-frequency line spectrum vibration and noise control methods for ship grillage structure are different. One is a general design idea to control the flexural wave fluctuation characteristics in the structure in general, and the other is an artificially optimized regulation of the band gap bandwidth range for local structural parameter optimization.

**Figure 8:**
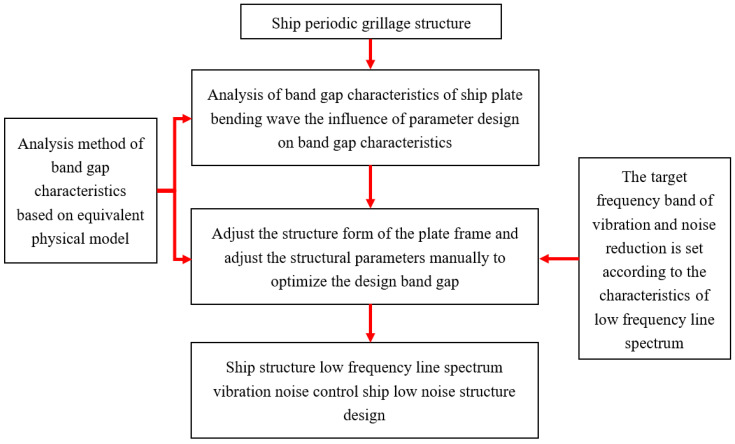
Ship hull grillage structure low noise structure design based on bandgaps characteristics.

Therefore, low-frequency line spectrum noise in ship structures is controlled by combining a global bandgap design approach with localized optimization techniques. This approach first determines the approximate bandgap location, then optimizes the flexural wave bandgap frequency range through local parameter adjustments and finally regulates low-frequency line spectrum vibrations and noise in the target frequency band via the flexural wave bandgap characteristics. This combined strategy forms an optimized design workflow that achieves directional vibration control through bandgap properties while offering new insights for low-noise structural design. The complete method and design process are shown in Figure 8.

### 3.4. Low Frequency Line Spectrum Vibration and Noise Control of Ship Grillage Structure Using Curved Wave Bandgap Characteristics

By applying a bandgap-based design approach, this study controls low-frequency line spectrum noise in ship grillages through flexural wave bandgaps. A comparative analysis of an original structure and its optimized counterpart verifies the method’s efficacy, offering a new strategy for low-noise structural design.

**Figure 9:**
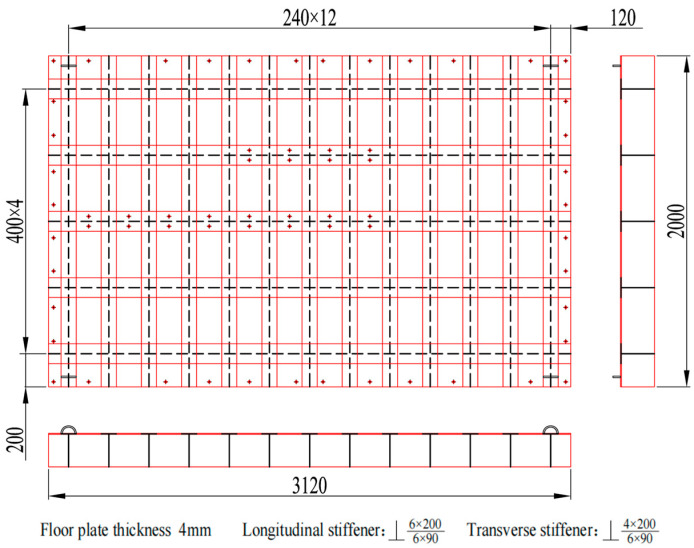
Schematic diagram of the original design scheme ship grillage structure. (The black dashed line represents the web; The inner red lines are paired vertically and horizontally, forming longitudinal and transverse stiffeners respectively; The outermost red line denotes the bottom plate; The dots indicate screw holes).

The original ship grillage structure measures 3120 mm in length, 2000 mm in width, and 200 mm in height. It contains five longitudinal stiffeners and thirteen transverse stiffeners, with uniform spacings of 400 mm and 240 mm, respectively. All stiffeners are welded T-profiles. The longitudinal stiffeners have a flange width of 120 mm and a web height of 200 mm, both with a thickness of 6.0 mm. Similarly, the transverse stiffeners feature a 90 mm wide flange and a 200 mm high web, also with a 6.0 mm thickness. The grillage plate itself has dimensions of 3120 × 2000 × 6.0 mm, as shown in Figure 9. The stiffeners and grillage structure are all low carbon steel. The section properties of longitudinal and transverse stiffeners of the original grillage structure are shown in Table 1.

**Figure 10:**
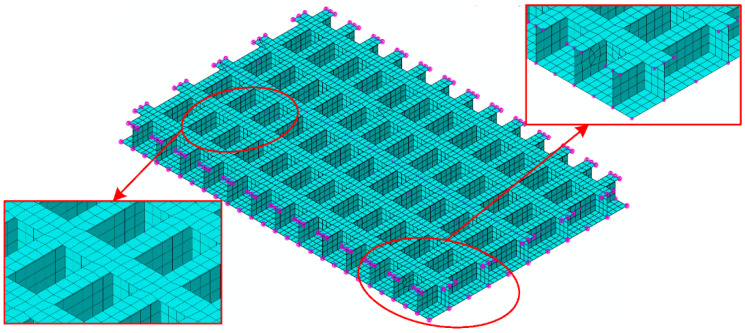
Numerical models of the original design scheme ship grillage structure.

**Figure 11:**
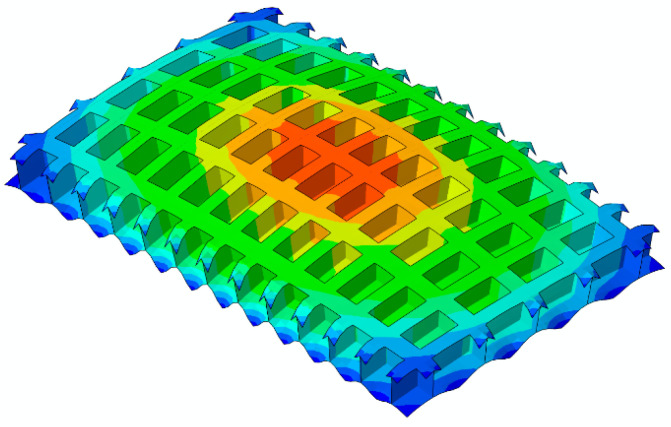
Low frequency beam-type flexural vibration modes of the original design scheme ship grillage structure (the 1 × 1 order beam flexural vibration mode of the original grillage structure f1 = 125.46 Hz).

The numerical model of the original grillage structure as shown in Figure 10 was established in Abaqus 2022. Considering the test verification of the subsequent model, the support condition was set as a four-sided elastic support on the four sides of the finite element model. After finite element analysis of the numerical model of the original grillage structure, the low-frequency flexural fluctuation characteristics of the original ship grillage structure are obtained. The low-frequency beam flexural vibration modes existing at 125.46 Hz are extracted, as shown in Figure 11. The equivalent calculation model parameters of the original grillage structure were calculated and shown in Table 2.

**Figure 12:**
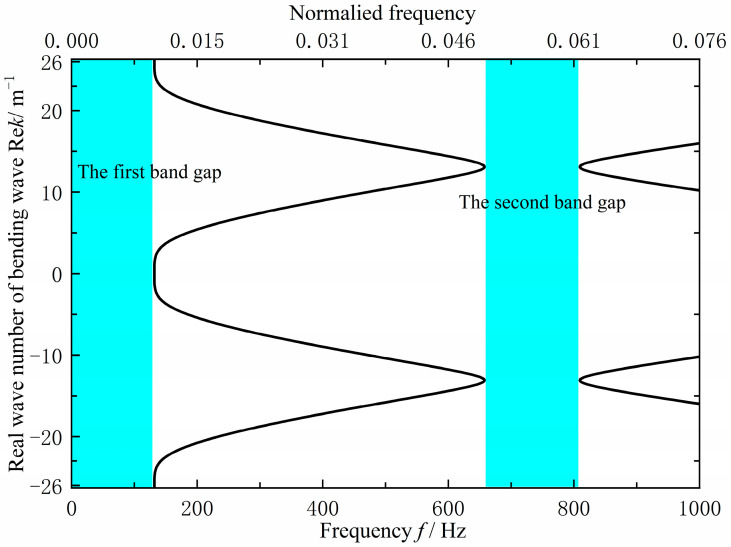
Flexural wave band gaps characteristics of the original design scheme grillage structure.

As shown in Figure 12, the original grillage structure exhibits flexural wave bandgaps in the ranges of 0–131.8 Hz and 657.6–809.4 Hz. However, these bandgaps do not adequately cover the excitation frequency band of the mechanical equipment or the target frequency range for noise control, the cutoff frequency of the first is too low, while the second is too high, resulting in limited suppression effectiveness. To improve performance, the structural configuration and parameters were optimized using a bandgap-based design approach. This adjustment increased the cutoff frequency of the first bandgap to approximately 200 Hz and shifted the second bandgap downward by about 150 Hz, thereby achieving better alignment with the target control frequencies.

Parameter analysis shows that transverse stiffeners mainly affect the first flexural wave bandgap, and longitudinal stiffeners the second. To raise the first bandgap’s cutoff frequency and shift the second to a lower range, we strengthen the longitudinal stiffeners while reducing the transverse ones. This is achieved by switching from a transverse-skeleton to a longitudinal-skeleton layout. Combining overall bandgap design with local parameter tuning allows us to adjust the bandgaps effectively for targeted frequency vibration control and low-noise design.

**Figure 13:**
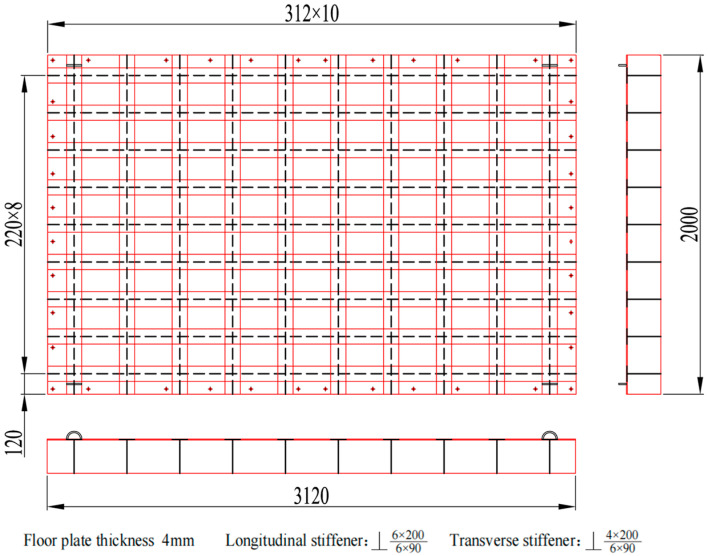
Schematic diagram of the improved design scheme ship grillage structure. (The black dashed line represents the web; The inner red lines are paired vertically and horizontally, forming longitudinal and transverse stiffeners respectively; The outermost red line denotes the bottom plate; The dots indicate screw holes).

**Figure 14:**
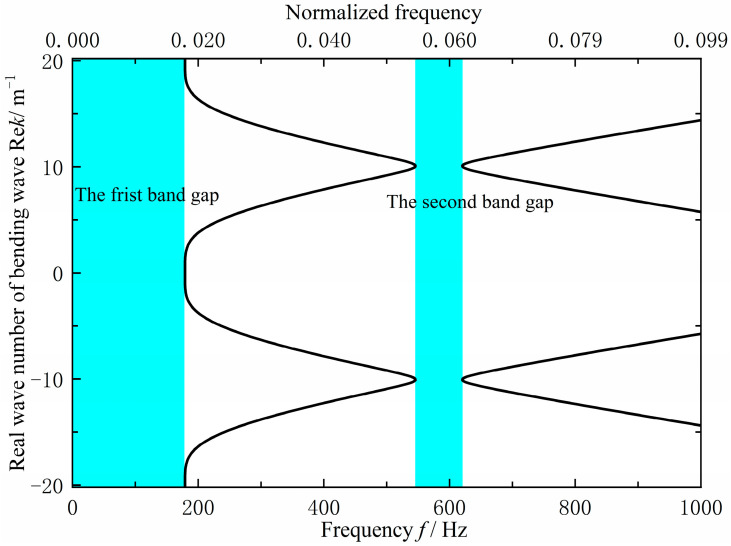
Flexural wave band gaps characteristics of the improved design scheme grillage structure.

The specific improvement size is 3120 × 2000 × 200 mm by reducing the transverse stiffeners from 13 to 10 and increasing their spacing to 312 mm. To achieve the target band control and low-noise objectives, bandgap calculations identified the optimal parameters: $EI*L=1.62\times 10^{6}\;N/m^{2}$, m∗L=750 kg/m, $k_{s}=1.75\times 10^{8}\;N/m$, $m_{s}=66\;kg$ and a=0.312 m. As shown in Figure 13, The final design uses a 220 mm longitudinal stiffener spacing and a 312 mm transverse spacing, with a 90 mm longitudinal panel width and a 4 mm transverse web thickness. Analysis indicates that the structure exhibits two effective flexural wave bandgaps in the 0–1000 Hz frequency range, specifically at 0–179.0 Hz and 545.8–620.3 Hz, as shown in Figure 14.

As can be seen from Figure 14, the improved grillage structure successfully raises the first bandgap’s cutoff frequency and shifts the second bandgap to a lower range. Meanwhile, according to the data in Table 3, the first bandgap widens by 47.2 Hz, ending at 179 Hz, while the second bandgap shifts down, reducing its center frequency by 150.45 Hz. These adjustments suppress line-spectrum vibrations in the target bands, lowering vibration peaks and meeting the design objective. This method is thus suitable for low-noise ship structure design.

### 3.5. Verification of Low-Frequency Line Spectrum Vibration and Noise Control Test for Ship Grillage Structure

**Figure 15:**
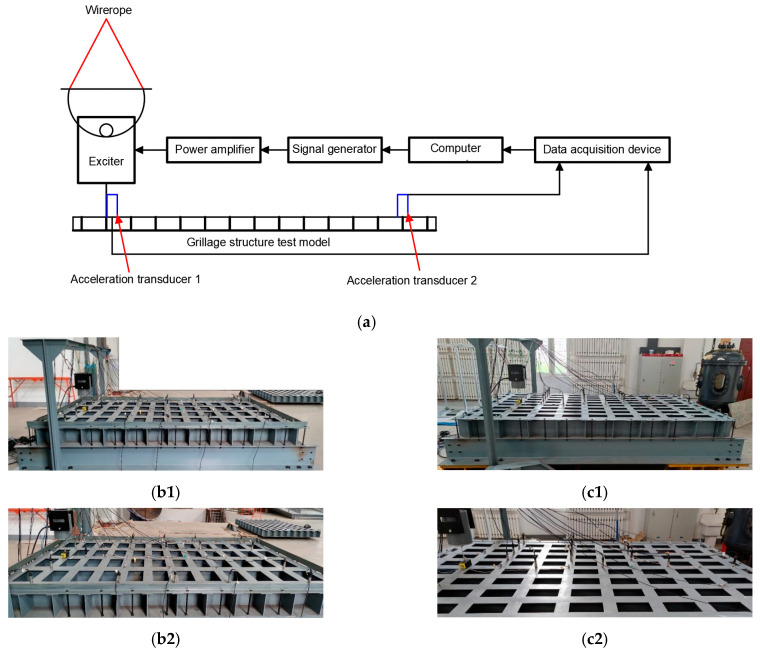
Implementation scheme of vibration signal measurement in verification test. (**a**) The schematic diagram of the vibration signal acquisition process for the verification test. (**b1**) The physical drawing of the original grillage structure. (**b2**) Excitation points and measuring points of the original grillage structure. (**c1**) Improved grillage structure physical drawing. (**c2**) Improve the excitation points and measuring points of the grillage structure.

Aiming at the research on noise control of ship grillage structures with gap characteristics, verifying the low-frequency noise control effectiveness of improved grillage structures, further investigating the gap characteristics of ship grillage structures, and designing vibration tests to validate the grillage structure. As shown in Figure 9 and Figure 13, experimental models of the original structure and the improved structure were fabricated. The experimental setup is shown in Figure 15. The test procedure involves a computer generating excitation signals and outputting them to a signal generator. These signals are amplified by a power amplifier to drive the shaker, which is directly connected to the experimental model. Vibration loads are applied to the model surface, and the model response is captured by accelerometers placed at various measurement points. The data are transmitted to a data acquisition device. Finally, the data are returned to the computer for storage and analysis. The structure is fixed by four supports, with a vibration table installed at the left end and accelerometers arranged at both ends. A vertical sweep excitation with a frequency range of 20–1000 Hz and a step interval of 5 Hz is applied. By analyzing the vibration signals, the propagation and attenuation characteristics of flexural waves are examined, with a particular focus on vibration suppression effects within the bandgap frequency range.

**Figure 16:**
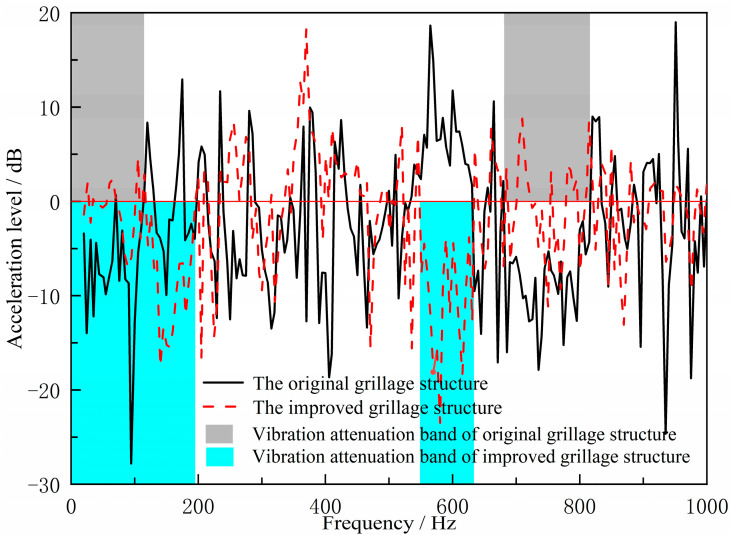
Flexural wave vibration attenuation characteristics in the original design scheme ship grillage structure and improved design scheme ship grillage structure.

**Figure 17:**
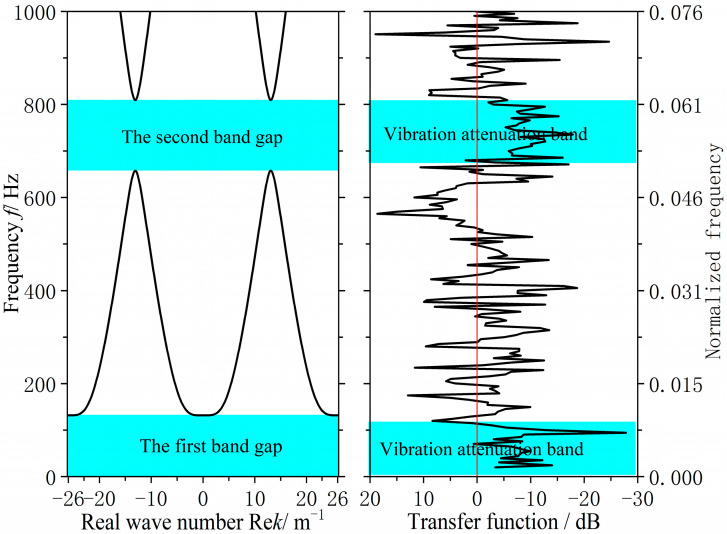
Flexural wave band gaps characteristics and vibration attenuation characteristics in the original design scheme ship grillage structure.

**Figure 18:**
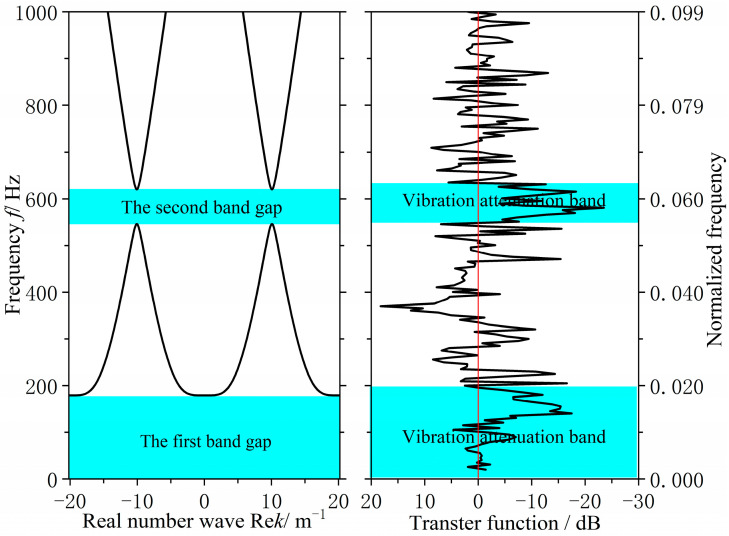
Flexural wave band gaps characteristics and vibration attenuation characteristics in the improved design scheme ship grillage structure.

Following the aforementioned validation tests, the measured acceleration signals underwent FFT transformation to obtain acceleration spectra. Based on analytical requirements, the displacement frequency response function was calculated via frequency domain integration. This yielded the bending vibration transfer functions for both the original hull grillage structure and the improved grillage structure, as shown in Figure 16. There are two significant vibration attenuation bands in both the original grillage structure and the improved grillage structure obtained from the test and analysis. Figure 17 and Figure 18, respectively, compare the flexural band gap characteristics of the original grillage structure and the improved grillage structure and the vibration attenuation characteristics obtained from the test analysis. Table 3 compares and analyzes the errors of the flexural band gap characteristics of the grillage structure and the corresponding vibration attenuation characteristics.

According to Figure 16, Figure 17 and Figure 18 and Table 4, the flexural wave bandgap ranges of both the original and improved lattice structures show good agreement with the measured vibration attenuation bands, experimentally validating the bandgap characteristics predicted by the equivalent computational model proposed in this study. To thoroughly verify the validity of the bandgap prediction, Figure 17 compares the theoretical simulation results of the predicted bandgap characteristics for the original structure Figure 12 with the corresponding experimental results of partial vibration transmission loss Figure 16. Figure 18 contrasts the theoretically predicted bandgap characteristics simulation results for the improved structure Figure 14 with the corresponding experimental vibration transmission loss results from Figure 16. These two sets of comparisons collectively demonstrate excellent agreement between theoretical predictions and experimental measurements within the bandgap frequency range, thereby validating the reliability of the equivalent computational model and bandgap design methodology employed.

Compared to the original structure, the modified lattice design exhibits a broader first bandgap and a lower-frequency shift in the second bandgap. Most notably, the target frequency band aligns perfectly with the optimized bandgap range, validating the effectiveness of the bandgap-based design methodology. These results indicate that structural modifications can selectively adjust flexural wave bandgap characteristics to achieve specific vibration control objectives. It should be noted, however, that this improvement does not manifest as an overall reduction in vibration amplitude across all frequencies. As shown in Figure 16, alterations to structural parameters modify dynamic characteristics, causing peak responses in the new vibration curve to shift in frequency rather than disappear. Within the target bandgap range, low-frequency line spectrum vibrations have been significantly attenuated, confirming the bandgap mechanism’s ability to effectively suppress noise in the targeted frequency range. The bending vibration transfer function obtained under unit excitation force reflects the structure’s inherent bandgap characteristics and vibration damping performance. In practical ship applications, when excitation is primarily dominated by specific mechanical frequencies, bandgap-based vibration reduction schemes hold promise for achieving more pronounced vibration and noise suppression effects.

Thus, the approach of controlling low-frequency line spectra through flexural wave bandgap tuning has been experimentally validated, providing a crucial strategy for low-noise ship structure design.

### 3.6. Low Noise Structural Design of Ship Cabin Using Flexural Wave Band Gap Characteristics

**Figure 19:**
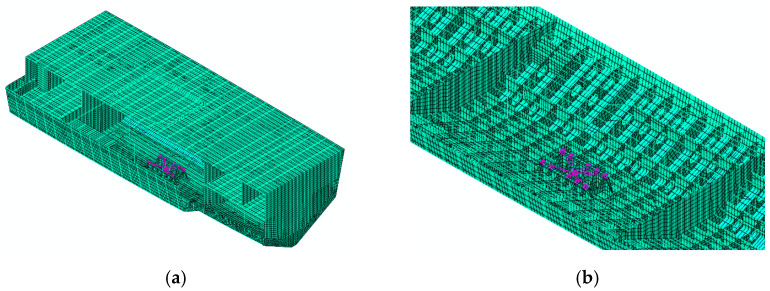
Ship engine room structure. (**a**) Cabin section structure. (**b**) Double layered bottom of cabin structure.

**Figure 20:**
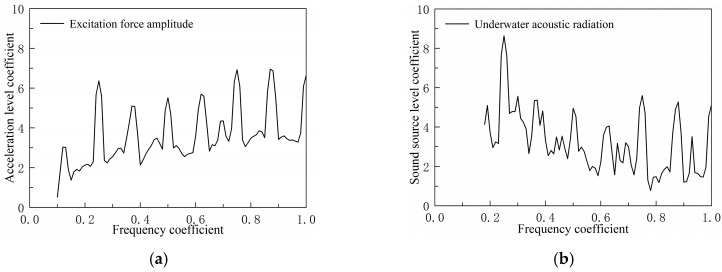
Mechanical equipment excitation force spectrum characteristics and underwater acoustic radiation spectrum characteristics curve. (**a**) Equipment excitation force load spectrum curve. (**b**) Underwater acoustic radiation spectrum curve.

The aforementioned ship deck vibration control method based on flexural wave bandgap characteristics can effectively control low-frequency vibration noise. Taking a certain type of ship compartment as the object, a machine room structural model was established as shown in Figure 19. An excitation force was applied to the base to simulate equipment loads, and the underwater sound radiation spectrum characteristics were analyzed, with the results shown in Figure 20.

As shown in Figure 20, underwater acoustic radiation exhibits significant peaks at the excitation force line spectrum frequency points, with the peak at the frequency coefficient of 0.25 being the most prominent. Therefore, the bandgap characteristics of the double-bottom structure were optimized specifically for this frequency band to control low-frequency line spectrum noise and achieve a low-noise structural design for the engine room structure.

By adopting a low-frequency line spectrum vibration noise control method based on periodic structural bandgap characteristics, the double-layer bottom grillage structure was optimized through reinforcing longitudinal beams, weakening transverse solid ribs, and partially replacing them with frame ribs.

**Figure 21:**
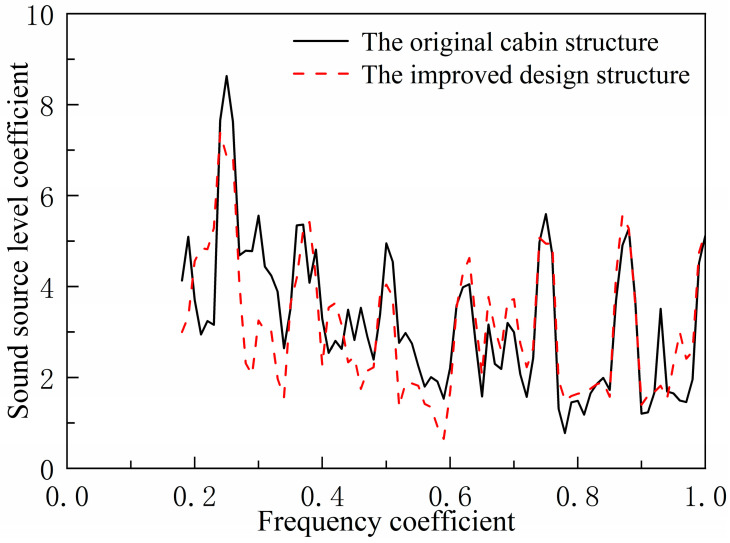
Comparison of underwater acoustic radiation spectrum characteristics.

Figure 21 compares the acoustic radiation spectra of the original compartment structure and the improved design. The improved structure exhibits significantly reduced line spectrum peaks near the 0.25 frequency, with peaks near 0.5 and 0.75 frequencies also showing a decreasing trend. Calculations indicate an 8.2 dB reduction in the total sound level of the underwater acoustic radiation source for the improved structure. This validates the effectiveness of the low-frequency line spectrum vibration noise control method based on bending wave bandgap characteristics and the low-noise structural design proposed herein, offering a new practical solution for low-noise ship structures.

## 4. Conclusions

In this study, a low-frequency line spectral vibration control method for ship grillage structures based on flexural wave bandgap modulation is proposed and validated. By establishing a theoretical model of periodic spring-vibrator coupled beams, the bandgap characteristics of the ship grillage structure are successfully predicted, which provides a theoretical basis for low-frequency vibration control.

The study adopts a combination of multiscale numerical simulation and experimental validation, and confirms that the periodic reinforcing bar structure can generate an effective flexural wave bandgap, which mainly forms two significant vibration attenuation bands of 0–179 Hz and 545.8–620.3 Hz. The experimental results show that the method achieves a vibration noise suppression effect of 8.2 dB in the target frequency band near 31.4 Hz, which verifies the accuracy of the theoretical prediction. This result provides a new technical way for the low-noise design of ships and has important engineering application value.

## Figures and Tables

**Table 1:** Parameters related to the properties of the reinforcement section of the original grillage structure.

Item	Unit	Longitudinal Stiffener	Transverse Stiffener
moment of inertia of cross-section *I*	m^4^	8.77621 × 10^−6^	7.95256 × 10^−6^
sectional area *A*	m^2^	1.92 × 10^−3^	1.74 × 10^−3^
mass per unit length	kg/m	15.072	13.659

**Table 2:** Equivalent computational model parameters of the original design scheme ship grillage structure.

Item	Symbol	Unit	Numerical Value
Equivalent bending stiffness	${EI}_{L}^{*}$	N·m^2^	1.3555 × 10^6^
Equivalent line quality	$m_{L}^{*}$	kg/m	852.1559
Stiffness of spring vibrator	*k*_s_	N/m	1.87025 × 10^8^
Mass of spring vibrator	*m*_s_	kg	66.08594
Bone spacing	*a*	m	0.24
Young’s modulus	*E*	Pa	2.1 × 10^11^
Poisson’s ratio	*v*	/	0.3
Density	*ρ*	kg/m^3^	7850

**Table 3:** Comparison of flexural wave band gaps characteristics between the original design scheme structure and the improved design scheme structure.

Item		The First Band Gap			The Second Band Gap		
		Initial Form Frequency	Cut-Off Frequency	Bandwidth	Initial Form Frequency	Cut-Off Frequency	Bandwidth
The original grillage structure	Hz	0	131.8	131.8	657.6	809.4	151.8
Improved grillage structure	Hz	0	179.0	179.0	545.8	620.3	74.5
Differentials	Hz	0	47.2	47.2	−111.8	−189.1	−77.3
Error		0.00%	35.81%	35.81%	−17.00%	−23.36%	−50.92%

**Table 4:** Comparison of flexural wave band gaps characteristics and vibration transmission attenuation characteristics.

Item		The Original Grillage Structure			Improved Grillage Structure		
		Initial Form Frequency	Cut-Off Frequency	Bandwidth	Initial Form Frequency	Cut-Off Frequency	Bandwidth
The first band gap	Hz	0	131.8	131.8	0	179.0	179.0
The first vibration attenuation band	Hz	0	115.0	115.0	0	195.0	195.0
Error		0.00%	−12.75%	−12.75%	0.00%	8.94%	8.94%
The second band gap	Hz	657.6	809.4	151.8	545.8	620.3	74.5
The second vibration attenuation band	Hz	685	820	135	550.0	635.0	85
Error		4.17%	1.31%	−11.07%	0.77%	2.37%	14.09%

## Data Availability

The original contributions presented in the study are included in the article. Further inquiries can be directed to the corresponding author.

## References

[1] Wu W. (2021). Research on the Analysis Method of Ship Vibration and Noise Transmission Path.

[2] Zhang L. (2013). Analysis and Experiment of Ship Vibration Sound Transmission Path under Operating Conditions. J. Huazhong Univ. Sci. Technol. (Nat. Sci. Ed.).

[3] Redondo J., Godinho L., Staliunas K., Sánchez-Pérez J.V. (2023). An Equivalent Lattice-Modified Model of Interfering Bragg Bandgaps and Locally Resonant Stop Bands for Phononic Crystal Made from Locally Resonant Elements. Appl. Acoust..

[4] Jing J., Sun P., Wu Z., Li F. (2025). Investigation on Enhanced Band-Gap Properties of 2D Hierarchical Phononic Crystals. Mech. Syst. Signal Process..

[5] Yang H., Cheng S., Li X., Yan Q., Wang B., Xin Y., Sun Y., Ding Q., Yan H., Li Y. (2023). Study on Bandgap and Vibration Attenuation Mechanism of Novel Chiral Lattices. Phys. B Condens. Matter.

[6] Bao Y., Jia Z., Tian Q., Luo Y., Zhang X., Kang Z. (2025). Phononic Crystal-Based Acoustic Demultiplexer Design via Bandgap-Passband Topology Optimization. Compos. Struct..

[7] Bao Y., Yao Z., Zhang Y., Hu X., Liu X., Shan Y., He T. (2024). Ultra-Broadband Gaps of a Triple-Gradient Phononic Acoustic Black Hole Beam. Int. J. Mech. Sci..

[8] Xiao Y., Shen W., Zhu H., Du Y. (2024). An Acoustic Black Hole Absorber for Rail Vibration Suppression: Simulation and Full-Scale Experiment. Eng. Struct..

[9] Gao N., Wang B., Lu K., Hou H. (2021). Complex Band Structure and Evanescent Bloch Wave Propagation of Periodic Nested Acoustic Black Hole Phononic Structure. Appl. Acoust..

[10] Yao X., Ji F. (2009). Research on the Transmission Characteristics of Vibration Waves in Typical Ship Structures. Vib. Shock.

[11] Liu J., Jin X. (2003). Propagation of Flexural waves in Periodic Stiffened Grillages. Noise Vib. Control.

[12] Lu L. (2017). Scattering of Flexural Waves by an Inhomogeneity in a Thin Plate. Wave Motion.

[13] Sorokin S.V., Ershova O.A. (2005). Analysis of the Energy Transmission in Compound Cylindrical Shells with and without Internal Heavy Fluid Loading by Boundary Integral Equations and by Floquet Theory. J. Sound Vib..

[14] Ruzzene M., Tsopelas P. (2003). Control of Wave Propagation in Sandwich Plate Rows with Periodic Honeycomb Core. J. Eng. Mech..

[15] Shen H., Wen J., Païdoussis M.P., Yu D., Asgari M., Wen X. (2012). Control of Sound and Vibration for Cylindrical Shells by Utilizing a Periodic Structure of Functionally Graded Material. Phys. Lett. A.

[16] Kafesaki M., Sigalas M.M., García N. (2000). Frequency Modulation in the Transmittivity of Wave Guides in Elastic-Wave Band-Gap Materials. Phys. Rev. Lett..

[17] An X., Yuan X., Sun G., He W., Lai C., Hou X., Fan H. (2024). Sandwich Plate-Type Metastructures with Periodic Graded Resonators for Low-Frequency and Broadband Vibration Attenuation. Ocean. Eng..

[18] Chang B., Wang S., Liang G., Liu Q., Xiao Y. (2025). Broadband Low-Frequency Diffuse Sound Transmission Loss of Multilayer Composite Plate-Type Metamaterials. Compos. Part C Open Access.

[19] Kong W., Fu T. (2024). A Novel Butterfly Double-Panel Metastructure Filled with Porous Materials for Broadband Low-Frequency Sound Absorption. J. Build. Eng..

[20] Xu Y., Hong Y., Li M., He X. (2023). Underwater Low-Frequency Sound Absorption Performance and Broadband Absorption Design of Membrane-Type Acoustic Metamaterials. Appl. Acoust..

[21] Zhang Y., Li X., Gai X., Xing T. (2025). Ultrathin Low-Frequency Tunable Sound Absorbing Structure Based on Buckling Plates. Appl. Acoust..

[22] Chen D., Li Y., Pan Z., Li X., Xu T., Li X. (2024). Low-Frequency Vibration Bandgaps and Deep Learning-Based Intelligent Design Method of Y-Shaped Core Sandwich Metabeams. Compos. Struct..

[23] Wang Y., Wang K.F., Wang B.L. (2024). Bandgap Estimation and Broadening of Piezoelectric Metamaterial Beams Undergoing Longitudinal Vibration. Smart Mater. Struct..

[24] Chen T. (1991). Ship Structural Mechanics.

[25] Kurzhakov I.Y. (1957). Ship Vibration.

